# Air Pollution and Estimated Health Costs Related to Road Transportations of Goods in Italy: A First Healthcare Burden Assessment

**DOI:** 10.3390/ijerph16162876

**Published:** 2019-08-12

**Authors:** Prisco Piscitelli, Barbara Valenzano, Emanuele Rizzo, Giuseppe Maggiotto, Matteo Rivezzi, Felice Esposito Corcione, Alessandro Miani

**Affiliations:** 1 Italian Society of Environmental Medicine (SIMA), 20149 Milan, Italy; 2 Euro Mediterranean Scientific Biomedical Institute (ISBEM), 1040 Bruxelles, Belgium; 3 Director of the Environmental Department of Apulia Region, 72100 Bari, Italy; 4 Translational Medicine PhD Candidate, University of Foggia, 71100 Foggia, Italy; 5 Former Director of the Institute of Motors, National Research Council (IM-CNR), 80100 Naples, Italy; 6 Department of Environmental Science and Policy, University of Milan, 20149 Milan, Italy

**Keywords:** road freight traffic, transportation of goods, air pollution, years of life lost, healthcare burden

## Abstract

*Background*: The Italian Society of Environmental Medicine has performed a preliminary assessment of the health impact attributable to road freight traffic in Italy. *Methods*: We estimated fine particulate matter (PM10, PM2.5) and nitrogen oxides (NOx) generated by road transportation of goods in Italy considering the number of trucks, the emission factors and the average annual distance covered in the year 2016. Simulations on data concerning Years of Life Lost (YLL) attributable to PM2.5 (593,700) and nitrogen oxides NO2 (200,700) provided by the European Environmental Agency (EEA) were used as a proxy of healthcare burden. We set three different healthcare burden scenarios, varying from 1/5 to 1/10 of the proportion of the overall particulate matter attributable to road freight traffic in Italy (about 7% on a total of 2262 tons/year). *Results*: Road freight traffic in Italy produced about 189 tons of PM10, 147 tons of PM2.5 and 4125 tons of NOx in year 2016, resulting in annual healthcare costs varying from 400 million up to 1.2 billion EUR per year. *Conclusion*: Road freight traffic has a relevant impact on air pollution and healthcare costs, especially if considered over a 10-year period. Any solution able to significantly reduce the road transportation of goods could decrease avoidable mortality due to air pollution and related costs.

## 1. Introduction

**Figure 1:**
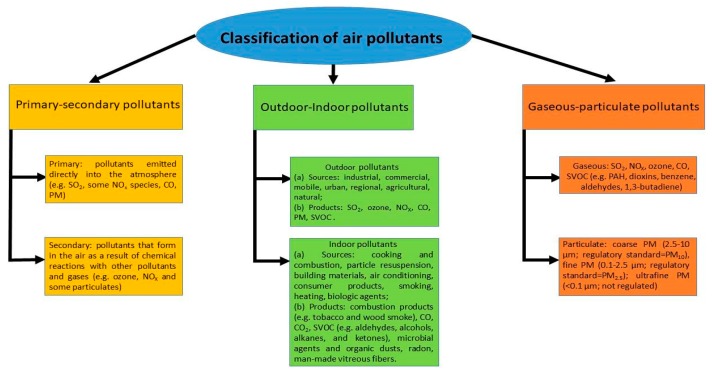
Classification of air pollutants (Modified from Bernstein et al. [39]).

**Figure 2:**
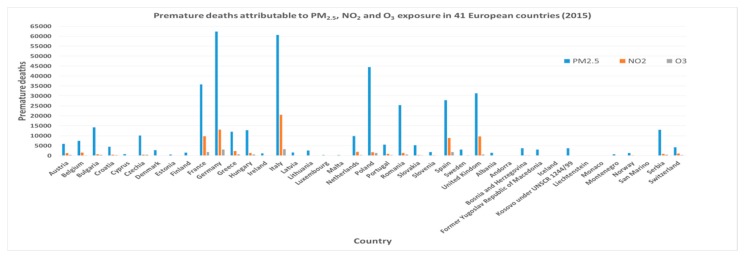
Premature deaths attributable to PM_2.5_, NO_2_ and O_3_ exposure in 41 European countries and the EU-28, 2015 (Modified from *Air quality in Europe—2018 report*, EEA, [24]).

Health is inextricably linked to environmental exposures and climate changes. According to the World Health Organization (WHO), air pollutants (Figure 1) represent a primary risk factor for human health [1]. This has been highlighted by several solid studies that have documented a significant association between air pollution and increased mortality from all causes [2,3,4,5,6,7,8], in particular for myocardial infarctions or cerebral-vascular accidents [9,10], as well as an increase in hospitalisations [11,12,13] even in the short-term for these conditions within 48–72 h of peak concentrations of fine particulate matter [14,15]. A significant proportion of respiratory diseases and lung cancers have also been found to be associated to air pollution [16,17,18,19,20,21,22]. The WHO has estimated that in about seven million of the premature deaths that occur every year are due to air pollution at an international level [23]. According to data from the European Environmental Agency (EEA), over 518,000 premature and avoidable deaths have been estimated to have occurred in Europe in the year 2015 (Figure 2), and at least 84,000 of those (16% of the total) occurred in Italy [24].

Fine dust pollution produces health impacts even at very low concentrations [1]. The EEA has documented that a total of 13% of the EU-28 urban population is exposed to fine particulate matter (PM_10_) levels above the daily limit value and approximately 42% is exposed to concentrations exceeding the stricter WHO Air Quality Guidelines (AQG) value for PM_10_ in 2016. Regarding fine particulate matter (PM_2.5_), 6% of the urban population in the EU-28 is exposed to levels above the European Union (EU) limit value, and approximately 74% is dealing with concentrations exceeding the WHO AQG value for PM_2.5_ in 2016 [24]. In fact, according to the 2005 WHO official guidelines, for the protection of human health we should refer to PM_10_ and PM_2.5_ thresholds that are reduced by 50%: annual average values not exceeding 20 μg/m^3^ for PM_10_ and 10 μg/m^3^ for PM_2.5_, compared to the current legal limits of 40 μg/m^3^ and 25 μg/m^3^ for PM_10_ and PM_2.5_, respectively [1].

Coal and fossil fuels are acknowledged as being most acknowledged responsible for the emission of PM_10_ and PM_2.5_ of anthropogenic origin in the atmosphere as well as other pollutants such as nitrogen dioxide (NO_2_) and sulfur dioxide (SO_2_), in addition to micropollutants and heavy metals. All these substances are persistent carcinogens and known to be dangerous for human health. PM_10_, PM_2.5_ and nitrogen oxides (NO_x_) are among the major air pollutants that are currently monitored at the European level. Domestic heating and the industrial sector contribute for a huge part of the total emission of these pollutants, but the way we deliver goods (i.e., via road freight traffic) both for the national and international market is supposed to have a relevant impact as well. According to Anenberg S. et al. [25], road traffic emissions would cause 385,000 premature deaths in the world (95% CI, 274,000–493,000): 5.38 deaths per 100,000 people in 2015. Global transportation-attributable deaths resulted in about 7.8 million of Years of Life Lost (YLL) in the year 2015, with 88% of these being caused by PM_2.5_.

Welfare costs associated with premature deaths attributable to road transportation were estimated approximately to reach approximately USD 1 trillion in 2015 and to represent 17% of total PM_2.5_- and ozone-related health costs [25]. Currently, there is an intense debate in Italy and throughout Europe concerning the opportunity of shifting a considerable proportion of transportation of goods from road to railway. On this basis, the Italian Society of Environmental Medicine has activated a specific working group to preliminarily assess the healthcare burden of air pollution attributable to road freight traffic in Italy.

## 2. Methods

We have referred to the calculation methodology developed for the COPERT model (*Computer Programme to calculate Emissions from Road Transport*) as implemented in the updated version issued in November 2007 (COPERT IV, EEA 2006) and currently used by the INEMAR (Air Emission Inventory), an official registry on emissions that collects data from the Italian regions that have data available [26,27,28,29,30,31,32,33]. First of all, we have indirectly estimated the amount of PM_10_, PM_2.5_ and NO_x_ generated by road freight traffic of goods in Italy for the year 2016. Our data derive from different official national datasets such as the Italian Vehicles Club/Public Registry of Vehicles (ACI/PRA), the Italian Registry of Air Emissions (INEMAR) that collects data from the Italian System of Regional Environmental Protection Agencies (ARPAs), and the Italian Institute for Statistics (ISTAT). The total amount of PM_10_, PM_2.5_ and NO_x_ generated by road freight traffic of goods in Italy was calculated using the following equation [26]:

*E = N × P × FE* (1)

where *E* indicates the emission we are looking for (PM_10_, PM_2.5_ and NO_x_ expressed in mg/year); *N* is the number of vehicles (*n* = 4,539,453) registered by the public registry of transportation ACI/PRA for the year 2016 [34]; *P* is the average annual distance covered by trucks in the year 2016 (282.36 km according to the National Institute for Statistics (ISTAT), representing the average value between 265.44 km estimated for national traffic and 299.28 Km estimated for international transport [35], and *FE* is the emission factor (mg/km). This latter value is estimated by INEMAR (the most updated data refer to the year 2014 and are based on the multiparametric model COPERT IV) [36] to be about 147.5 mg/km for PM_10_ (representing the average value between 77 mg/km estimated for vehicles < 3.5 tons, and 218 mg/km estimated for trucks > 3.5 tons); 114.5 mg/km for PM_2.5_ (average value between 60 mg/km for vehicles < 3.5 tons and 169 mg/km for trucks > 3.5 tons); and 3218 mg/km for NO_x_ (average value between 864 mg/km for vehicles < 3.5 tons, and 5572 mg/km for trucks > 3.5 tons).

After estimating the emissions, we have used the data provided by the 2018 European Environmental Agency Air Quality Report (based on 2015 figures) concerning the annual number of YLL attributable to PM_2.5_ and NO_2_ in Italy: 593,700 YLL for PM_2.5_ and 200,700 YLL for NO_2_ [24]. The National Emission Inventory (held by the Italian Institute for the Environment (ISPRA)) estimates that overall road freight traffic in Italy accounts for about 7% of the overall particulate matter (2262 ton/year) produced in Italy [37]. In order to assess the YLL attributable to these source-specific emissions, we have set three different scenarios varying approximatively from 1/5 to 1/10 of air pollution attributable to road freight transportation of goods. Thus, we assumed that the sum of PM_2.5_ and NO_x_ emissions would result in being responsible for 0.5%, 1% or 1.5% of total YLL in Italy, estimated by the EEA in 593,700 for PM_2.5_ and 200,700 for NO_2_, as already reported, for a total of 794,400 YLL. Years of Life Lost were used as a proxy of healthcare burden. The economic value used for the health costs estimation was computed according to the available literature concerning EU residents and assessed at 100,000 EUR per YLL [38].

## 3. Results and Discussion

We have estimated that the road freight transportation of goods in Italy produced approximately 189 tons of PM_10_, along with 147 tons of PM_2.5_ and 4125 tons of NO_x_ in the year 2016. In the first scenario, we have assumed that the PM_2.5_ and NO_x_ generated by road freight transportation of goods in Italy would be responsible for 0.5% of total YLL in Italy, estimated by the EEA in 593,700 for PM_2.5_ and 200,700 for NO_2_, for a total of 794,400 [24]. Similarly, in the second and third scenarios, we assumed that the same pollutants would account for 1% and 1.5% of total YLL in Italy, respectively (Table 1). Therefore, according to this first health impact assessment, the costs concerning the years of life lost attributable to road freight traffic of goods in Italy account for a minimum of 400 million EUR per year in the most favorable scenario, and up to 800 million and 1.2 billion EUR in the intermediate and worse scenarios, respectively.

Our study provides a first attempt to assess the healthcare burden and an estimation of costs attributable to the road transportation of goods in Italy. As a proxy of these costs, we have referred to the value of 100,000 per YLL [38], which is consistent with other estimations provided by different international institutions. Actually, in order to assess economic benefits deriving from the reduction of mortality risk, the U.S. Food and Drug Administration (FDA) and the U.S. Environmental Protection Agency (EPA) have estimated the Value of Statistical Life (VSL) in 2010 to range from 7.9 million to 9.1 million USD, respectively [39]. Similar estimations provided by the U.S. Department of Transportation assessed the VSL value to be between 9.2 (2014) and 9.6 million USD (2016) [40,41,42,43]. Moreover, if we consider a VSL of 9 million USD (that corresponds to about 8 million EUR) and we divide it by the average life expectancy (over 80 years old in Italy referring to the year 2015), we obtain a value of about 100,000 EUR per each year of life, being totally consistent with the available literature as well as the average cost computed in our analysis for each YLL.

This research has been carried out taking into account the available evidence concerning particulate matter emissions in Europe, although it has not been possible to include estimations concerning the age of vehicles and unofficial sources of information such as those obtained by projects based on diffuse measurements [44,45,46,47,48,49,50]. Our findings could also be regarded as conservative also because they consider the average annual distance covered by trucks registered in Italy but not in other countries, both for national and international freight traffic of goods. However, the estimations that underlie the simulations provided (provided by the National Agency for the Environment (ISPRA)) include the overall emissions of particulate matter attributable to road freight traffic with respect to the total particulate matter produced each year in Italy, without excluding the contribution coming from trucks registered abroad that pass through the Italian roads and thus impacting on the Italian population in terms of pollution consequences.

We have considered all categories of vehicles used for goods transportation (trucks, three-wheelers, quadricycles, trailers and semi-trailers), assuming that they were all circulating. Traffic itself can be divided into “diffuse traffic” (the urban one) and “linear traffic” (typical of the great traffic arteries: highways, tangential roads, expressways, state and provincial roads). We have considered road freight transportation of goods as being linear traffic due to the extreme difficulty of assessing its urban component and assuming a prevalence of the extra-urban component in the transportation of goods as confirmed by the average distance covered by trucks in our data sources (>200 km). Moreover, we have estimated only some tailpipe emissions (PM_2.5_, PM_10_ and NO_x_), excluding CO, SO_2_ and volatile organic compounds (VOCs), and without taking into account evaporative (produced even when vehicles are stopped or with the engine turned off) and PM_10_ emissions from abrasion, produced by friction from brakes, tires and asphalt. However, the latter two types of emissions are quantitatively important: as a consequence, our results could be underestimated. It must be also highlighted that our estimations used PM_2.5_ and NO_x_ as indicators of air pollution produced by road freight transportation of goods but did not consider possible independent effects of PM_10_ itself, PM_0.1_, SO_2_, CO and others. In our study, we have mainly referred to PM_2.5_, but it should not be forgotten that ultrafine particulate matter (with a PM of less than 0.1 micron), due to its small size, is able to penetrate into pulmonary interstices, causing inflammation and oxidative stress by passing into the blood circulation through the capillary alveolus barrier.

Moreover, to estimate health costs, we made an assumption in order to assess the YLL attributable to the source-specific emissions by setting three different scenarios varying approximatively from 1/5 to 1/10 of air pollution due to particulate matter attributable to road freight traffic (about 7% of the total particulate matter emissions at the national level) and assuming that the sum of PM_2.5_ and NO_x_ emissions would result in being responsible for 0.5%, 1% or 1.5% of total YLL in Italy (estimated by the EEA to 794,400 YLL for PM_2.5_ and NO_2_ exposures). Therefore, our findings could be considered conservative and may have produced additional underestimations of the phenomenon.

Many studies have confirmed that air pollutants have short-term influences on the risk of myocardial infarction. A research carried out by Schwartz, Dockery and Neas [51] has documented that an average increase of 10 μg/m^3^ of PM_2.5_, typically linked to combustion, is associated with a +1.5% increase (95% CI 1.1–1.9) of the total daily mortality, as well as with a +3.3% increase in deaths caused by Chronic Obstructive Pulmonary Diseases (COPD) and a +2.1% in deaths due to ischemic heart attacks within 48 h. Badaloni, Cesaroni et al. [2] have analysed the effects on non-accidental, cardiovascular (CVD) and ischemic heart disease (IHD) mortality due to population long-term exposure to PM_10_, PM_2.5_, PM_2.5_ absorbance and particulate matter components (copper, iron, zinc, sulfur, silicon, potassium, nickel, and vanadium) in a large cohort of 1,249,108 residents of Rome. Bhaskaran, Hajat et al. [52] have conducted a systematic review on the association between air pollution and changes in both general and cardiovascular mortality, selecting only 26 large studies (19 papers addressing short-term effects at 24 h from peaks of emissions and seven studies assessing long term effects) that had myocardial infarction as their primary objective with respect to exposure to particulate matter, black carbon, ozone, carbon monoxide, nitrogen oxides, sulfur dioxide and also vehicular traffic. These studies documented statistically significant detrimental effects of PM_2.5_ (increase in risk between 5 and 17% for each increase of 10 micrograms per cubic meter); PM_10_ (0.7–11% for each increment of 10 micrograms per cubic meter); CO (2–4% for each increment of 10 micrograms per meter cube) and NO (1–9% for each increment of 10 micrograms per cubic meter).

Similarly, the Health Effects of Particles on Susceptible Subpopulations Study (HEAPSS) Study (Health Effects of Particles on Susceptible Subpopulations Study) followed 27,000 people (aged 35–84 years; distributed in different cohorts) affected by myocardial infarction between 1992 and 2000 in five European cities (Augsburg in Germany, Barcelona in Spain, Helsinki in Finland, Rome in Italy and Stockholm in Sweden) monitoring PM_0.1_, PM_10_, NO_2_ and CO concentrations along with patient follow-up and concluded in favour of an immediate effect (within 24 h although not cumulative over the weeks) of exposure to air pollutant peak concentrations and occurrence of new episodes of acute myocardial infarctions, which were observed in 10% of the enrolled subjects. The study found strong associations between all exposure indicators and mortality. Exhaust gas pollutants emitted by vehicles were confirmed to play an important role in determining the excess mortality [53].

A large American study [54] in1980 has analysed the particulate air pollution data produced by combustion of fossil fuels in 151 metropolitan areas in the United States: 552,138 adult residents were followed until 1990. The study found an association between air pollution and mortality from lung cancer and cardiovascular or pulmonary causes in areas characterised by heavy environmental pressure compared to less polluted areas, finding statistically significant increases (+15% and +17%, respectively). The increased risk was not attributable to cigarette smoking. Some Dutch cohort studies seem to show that exposure to air pollution early in life might contribute to the development of asthma throughout childhood and adolescence [55] and that a long-term exposure can reduce life expectancy [56].

Norwegian studies conducted on over 16,000 subjects in their 40s living in Oslo [57] confirmed the effect of an association between air pollution and lung diseases (HR: 1.16 with 95% CI: 1.06–1.26), lung cancer (HR: 1.11 with CI: 95% 1.03–1.19; ischemic heart disease (HR: 1.08 with 95% CI: 1.03–1.12) and cerebrovascular diseases (HR: 1.04 with 95% CI, 0.94–1.15). The long-term effects of air pollution on mortality were also studied in 14,284 adults living in seven French cities in the frame of the PAARC Study (acronym for Air Pollution and Chronic Respiratory Diseases) which included daily measurements for three years of sulfur dioxide, atmospheric particulate, black smoke, nitrogen dioxide and nitrogen oxide and the application of Cox proportional risk models to control individual factor confounders (smoking, educational level, body mass index, occupational exposure). Even in this study, the excess of mortality due to lung cancer and cardiopulmonary causes for each increase of 10 µg/m^3^ was confirmed [58].

Several studies have documented a link between increased cardiovascular or respiratory mortality and exposure to fine particulate matter (PM_10_ and PM_2.5_) [9,10,11,12,20,21,22]. It has also been demonstrated that peaks of such air pollutants increase hospital admissions for cardiac or respiratory diseases, especially in vulnerable subgroups (e.g., asthmatic patients), as well as that chronic exposures result in a higher risk of cancer [59,60,61,62]. Moreover, the role of workers’ exposures should be taken into account, as should the specific case of children; both of which represent two populations that are characterised by greater susceptibility to acute and chronic exposures [63,64,65,66,67,68,69,70,71,72,73,74,75,76]. As for neoplastic diseases other than lung cancer, Magnani et al. [77] demonstrated the possibility of an association between childhood leukemia and traffic pollution. The results of the SETIL study provided further support concerning the association between childhood leukemia and exposure to vehicular traffic [78]. Moreover, a large cohort study carried out by Renzi M., Cerza F. and Gariazzo C. et al. [79] concluded that long-term exposure to NO_x_ and O_3_ could also be associated with diabetes. Our estimations suggest that new solutions should be adopted in transportation policies for goods in Italy, in order to reduce the impact of pollution by freight transport, e.g., through the use of modern rail transport or using modern engines, hybrids and hydrogen-powered trains [80].

## 4. Conclusions

Transportation of goods on Italian roads represents the fifth major source of particulate matter air pollution after domestic heating, intensive breeding, the industrial sector and urban traffic (light vehicles: cars, motorbikes), but before agriculture and energy production. Road freight traffic of goods has a relevant impact both in terms of air pollution and healthcare costs, especially if considered over a 10-year period or over several decades. Any solution able to significantly reduce the road transport of goods could therefore decrease avoidable mortality due to atmospheric pollution and related costs.

As stated by the Communication of the European Commission of 2 February 2000 [81], the precautionary principle stated by Article 191 of the Treaty on the Functioning of the European Union should be taken into account much more in order to ensure a high level of environmental and health protection through preventive decisions in case of risk. According to the Paris Agreement on climate change mitigation, the European Union has approved its long-term strategy to reach climate neutrality by the year 2050, including a 50% mid-term cut of current emissions. Moreover, the EU Union has already concluded the public consultation to support the Fitness Check of EU Ambient Air Quality Directive, which could result in incorporating the WHO thresholds for PM_10_ and PM_2.5_, corresponding to a 50% drop-out compared to the current European limits.

## Figures and Tables

**Table 1:** Health costs attributable to road freight traffic of goods.

	Costs Related to PM_2.5_	Costs Related to NO_2_	Total Costs (PM_2.5_ + NO_2_)
1st scenario Assuming road freight traffic of goods being responsible for 0.5% of Years of Life Lost (YLL) for PM_2.5_ and NO_2_ estimated by the EEA for Italy	0.5% × 593,700 YLL * × 100,000 € ** = 296,850,000 €	0.5% × 200,700 YLL * × 100,000 € ** = 100,350,000 €	397,200,000 €
2nd scenario Assuming road freight traffic of goods being responsible for 1% of Years of Life Lost (YLL) for PM_2.5_ and NO_2_ estimated by the EEA for Italy	1% × 593,700 YLL * × 100,000 € ** = 593,700,000 €	1% × 200,700 YLL * × 100,000 €** = 200,700,000 €	794,400,000 €
3rd scenario Assuming road freight traffic of goods being responsible for 1.5% of Years of Life Lost (YLL) for PM_2.5_ and NO_2_ estimated by the EEA for Italy	1.5% × 593,700 YLL * × 100,000 € ** = 890,550,000 €	1.5% × 200,700 YLL * × 100,000 € ** = 301,050,000 €	1,191,600,000 €

* 794,400 are the total Years of Life Lost (YLLs) due to PM_2.5_ and NO_2_ in Italy for the year 2015 [24]. ** 1 YLL = 100,000 EUR (average value [38]).
